# Efficacy and Safety of Video-Laryngoscopy versus Direct Laryngoscopy for Double-Lumen Endotracheal Intubation: A Systematic Review and Meta-Analysis

**DOI:** 10.3390/jcm10235524

**Published:** 2021-11-25

**Authors:** Katarzyna Karczewska, Szymon Bialka, Jacek Smereka, Maciej Cyran, Grazyna Nowak-Starz, Jaroslaw Chmielewski, Michal Pruc, Pawel Wieczorek, Frank William Peacock, Jerzy Robert Ladny, Lukasz Szarpak

**Affiliations:** 1 Department of Anesthesiology, Masovian Specialist Hospital, 26-617 Radom, Poland; k.karczewska@interia.eu; 2 Department of Anesthesiology and Intensive Care, Medical University of Silesia, 41-800 Zabrze, Poland; szymon.bialka@gmail.com; 3 Department of Emergency Medical Service, Wroclaw Medical University, 51-618 Wroclaw, Poland; Jacek.Smereka@umed.wroc.pl; 4 Research Unit, Polish Society of Disaster Medicine, 05-806 Warszawa, Poland; maciej.cyran@uczelniamedyczna.com.pl (M.C.); m.pruc@ptmk.org (M.P.); paulis.vesper@gmail.com (P.W.); 5 Institute of Outcomes Research, Maria Sklodowska-Curie Medical Academy, 03-411 Warsaw, Poland; 6 Institute of Health Sciences, Jan Kochanowski University of Kielce, 25-369 Kielce, Poland; gnowakstarz@ujk.edu.pl; 7 College of Rehabiliation, 01-234 Warsaw, Poland; jaroslaw.chmielewski@ios.home.pl; 8 Research Unit, Polonia University, 42-200 Czestochowa, Poland; 9 Henry JN Taub Department of Emergency Medicine, Baylor College of Medicine, Houston, TX 77030, USA; frankpeacock@gmail.com; 10 Department of Emergency Medicine, Bialystok Medical University, 15-295 Bialystok, Poland; ladnyjr@wp.pl; 11 Research Unit, Maria Sklodowska-Curie Bialystok Oncology Center, 15-027 Bialystok, Poland

**Keywords:** double-lumen tube, endotracheal intubation, one-lung ventilation, systematic review, meta-analysis, randomized controlled trials

## Abstract

The available meta-analyses have inconclusively indicated the advantages of video-laryngoscopy (VL) in different clinical situations; therefore, we conducted a systematic review and meta-analysis to determine efficacy outcomes such as successful first attempt or time to perform endotracheal intubation as well as adverse events of VL vs. direct laryngoscopes (DL) for double-lumen intubation. First intubation attempt success rate was 87.9% for VL and 84.5% for DL (OR = 1.64; 95% CI: 0.95 to 2.86; I^2^ = 61%; *p* = 0.08). Overall success rate was 99.8% for VL and 98.8% for DL, respectively (OR = 3.89; 95%CI: 0.95 to 15.93; I^2^ = 0; *p* = 0.06). Intubation time for VL was 43.4 ± 30.4 s compared to 54.0 ± 56.3 s for DL (MD = −11.87; 95%CI: −17.06 to −6.68; I^2^ = 99%; *p* < 0.001). Glottic view based on Cormack–Lehane grades 1 or 2 equaled 93.1% and 88.1% in the VL and DL groups, respectively (OR = 3.33; 95% CI: 1.18 to 9.41; I^2^ = 63%; *p* = 0.02). External laryngeal manipulation was needed in 18.4% cases of VL compared with 42.8% for DL (OR = 0.28; 95% CI: 0.20 to 0.40; I^2^ = 69%; *p* < 0.001). For double-lumen intubation, VL offers shorter intubation time, better glottic view based on Cormack–Lehane grade, and a lower need for ELM, but comparable first intubation attempt success rate and overall intubation success rate compared with DL.

## 1. Introduction

The increasing development of thoracoscopic procedures is inevitably associated with the use of single-lung ventilation. Such a strategy allows, on one hand, to expose the surgical field while maintaining ventilation of the non-operated lung [1]. One of several methods of providing single-lung ventilation, currently considered the gold standard, is the use of double-lumen endotracheal tubes (DLTs) [2]. The application of DLTs, due to their large diameter or the need for a rotational insertion technique, is associated with a significant risk of prolonged intubation, intubation failure, and airway trauma [3,4]. On the other hand, studies have confirmed that the use of video-laryngoscopes (VL), compared with traditional laryngoscopy, improves the visibility of anatomical structures during intubation [5]. VLs facilitate intubation with a traditional tube in cases of both “normal” and difficult airways [6]. However, the available meta-analyses have inconclusively indicated the advantages of VL in different clinical situations [7,8,9,10].

Therefore, we conducted this systematic review and meta-analysis to determine the efficacy outcomes such as successful first attempt and time to perform endotracheal intubation as well as adverse events of VLs vs. direct laryngoscopes (DL) for double-lumen intubation.

## 2. Materials and Methods

This systematic review and meta-analysis was performed in accordance with the criteria outlined in the Preferred Reporting Items for Systematic Reviews and Meta-Analyses (PRISMA) statement (Table S2) [11]. Due to the study design (meta-analysis), neither institutional review board approval nor patient informed consents were required.

### 2.1. Search Strategy

Two reviewers (KK and JS) independently performed a comprehensive literature search using the PubMed, Scopus, Web of Science, and Cochrane Central Register of Controlled Trials databases. The most recent search was conducted on 30 September 2021. Terms related to double-lumen endotracheal intubation were applied: “double-lumen”, “laryngoscopy”, “video-laryngoscopy”, and “direct laryngoscopy”. Additionally, we performed a manual search and review of the references listed in the retrieved articles. All references were saved in an EndNote (EndNote, Inc., Philadelphia, PA, USA) library used to identify duplicates.

### 2.2. Study Selection Criteria

The studies included in this meta-analysis met the following PICOS criteria: (1) Participants: intubated adult patients; (2) Intervention: endotracheal intubation by using a DLT with video-laryngoscopes; (3) Comparison: endotracheal intubation by using a DLT with Macintosh direct laryngoscope; (4) Outcomes: detailed information concerning first intubation attempt success rate, overall intubation success rate, intubation time, glottic view, external laryngeal manipulation (ELM), and adverse events; and (5) Study design: randomized controlled trials. The exclusion criteria were as follows: (1) studies involving pediatric patients; (2) non-RCTs; (3) editorials; (4) conference abstracts; and (5) guidelines.

### 2.3. Data Extraction

Two reviewers (MC and KK) independently extracted data from the identified eligible studies by using a specifically designed data extraction form in Microsoft ExcelTM (Microsoft Corp., Redmond, WA, USA). Another author cross-checked these data before analysis (LS).

The following data were extracted from each study: first intubation attempt success rate, overall intubation success rate, intubation time, glottic view, and ELM. We also analyzed the adverse events that occurred during tracheal intubation. Where there were suspected discrepancies in the data, we contacted the relevant authors directly.

### 2.4. Risk of Assessment Bias

We applied a revised tool to assess the risk of bias in randomized trials (RoB-2) [12] to evaluate the risk of bias of the included studies. The RoB-2 tool examines five bias domains: (1) bias arising from the randomization process; (2) bias due to deviations from intended intervention; (3) bias due to missing outcome data; (4) bias in the measurement of the outcome; and (5) bias in the selection of the reported results. The risk of bias in each of these domains can be rated as “high”, “some concerns”, or “low”. The overall RoB-2 judgment at the domain and study level was attributed in accordance with the criteria specified in the RobVis tool [13]. The risk of bias was determined independently by two reviewers (KK and MC); disagreements were resolved by a third reviewer (LS) if necessary.

### 2.5. Statistical Analysis

The meta-analysis was entirely conducted with Review Manager software, version 5.4 (Nordic Cochrane Centre, The Cochrane Collaboration, Copenhagen, Denmark) and the Stata 14 software (StataCorp. LP, College Station, TX, USA). The significance level for all statistical tests was *p* <  0.05 (two-tailed). For dichotomous data, we used odds ratios (ORs) as the effect measure with 95% confidence intervals (CIs), and for continuous data, we applied mean differences (MDs) with 95% CI. When a continuous outcome was reported in a study as median, range, and interquartile range, we estimated means and standard deviations with the use of a formula described by Hozo et al. [14]. For meta-analysis, we utilized the random effects model (assuming a distribution of effects across studies) to weigh estimates of studies in proportion to their significance [15]. Heterogeneity was determined with the I^2^ statistic, in which the results ranged from 0% to 100%. Heterogeneity was interpreted as not observed when I^2^ = 0%, low when I^2^ = 25%, medium when I^2^ = 50%, and high when I^2^ = 75% [16,17].

Subgroup analyses were performed on first intubation success rate, overall success rate, time to intubation; glottic view, and ELM were investigated by separating the VL types. Videolaryngoscopes, depending on the construction, were divided into the following groups: Macintosh blade VLs, channeled VLs, and video-tubes and scopes.

## 3. Results

### 3.1. Characteristics of Studies Included in the Meta-Analysis

**Figure 1:**
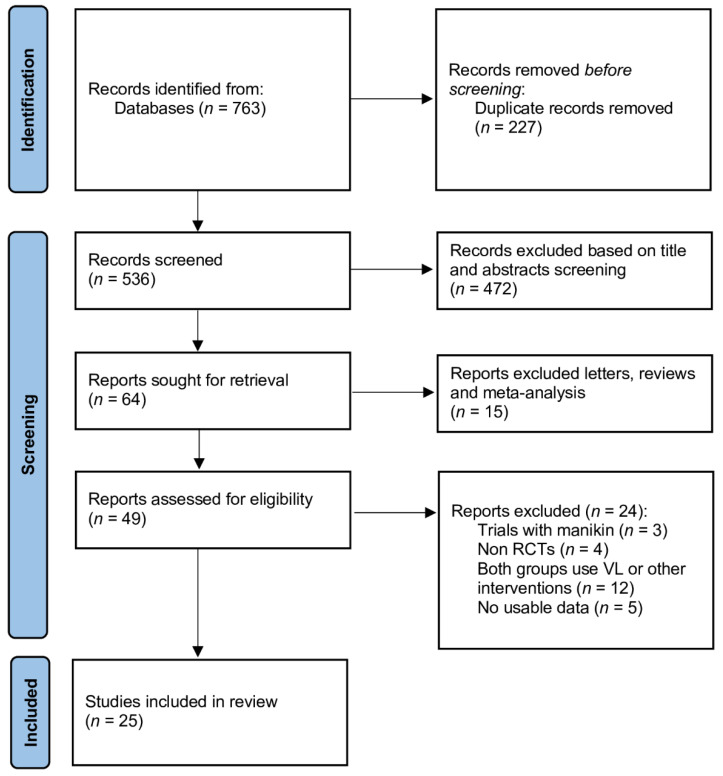
Database search and selection of studies according to PRISMA guidelines.

The initial literature search yielded 761 citations, and further two citations were identified through manual citation and a reference search of relevant articles (Figure 1). After excluding duplicate studies, 539 articles remained. After screening the titles and abstracts of all the retrieved articles, 473 papers were excluded. Then, the full texts were reviewed, and 32 studies were excluded because they did not involve Macintosh direct laryngoscope as a comparator group, consisted of manikin trials, involved different interventions, or were reviews or meta-analyses. Ultimately, 25 studies [18,19,20,21,22,23,24,25,26,27,28,29,30,31,32,33,34,35,36,37,38,39,40,41,42] published in the period of 2010–2021 were included in this meta-analysis.

These 25 studies included 2154 adult patients (Table 1 and Table S1). A detailed characterization of the patients in the VL and DL groups is presented in Supplementary Digital File in Table S3 and Figures S1 and S2. Each study was screened for risk of bias and for methodological quality with the Cochrane Collaboration’s tool for assessing the risk of bias (Figures S5 and S6). All studies met the criteria for high-quality randomized controlled trials.

### 3.2. First Intubation Attempt Success Rate

A total of 23 studies reported the first intubation attempt success rate. Pooled analysis revealed the first attempt success rate of 87.9% in VL compared with 84.5% for DL (OR = 1.64; 95% CI: 0.95 to 2.86; I^2^ = 61%; *p* = 0.08).

**Figure 2:**
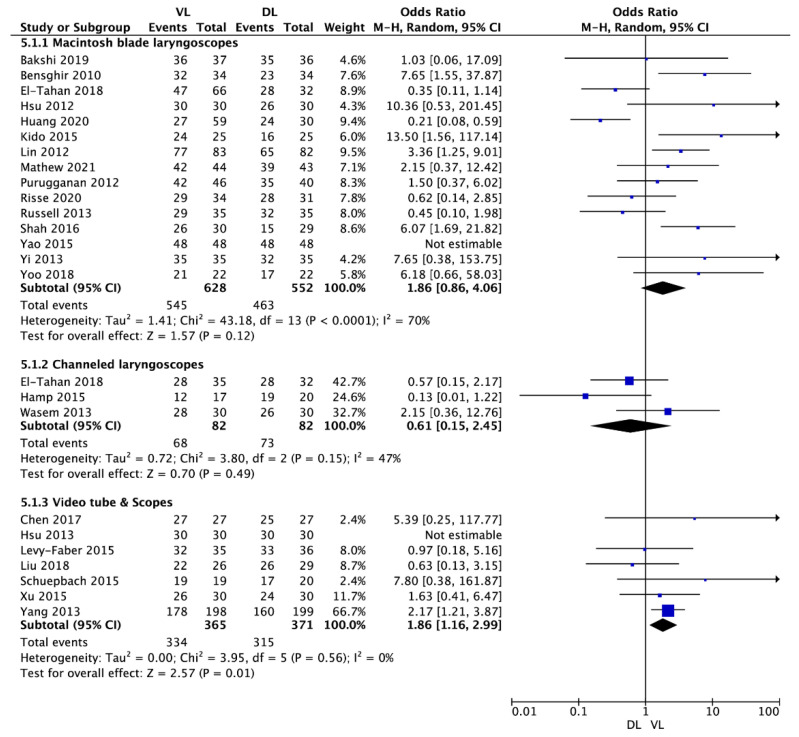
Forest plot of first intubation attempt success rate among video-laryngoscope and direct-laryngoscope groups. The center of each square represents the weighted odds ratios for individual trials, and the corresponding horizontal line stands for a 95% confidence interval. The diamonds represent pooled results. Legend: CI = confidence interval; DL = direct laryngoscopy; OD = odds ratio; VL = video-laryngoscopy.

Subgroup analysis showed that the first intubation success rate for VL with a Macintosh shape blade compared with the standard Macintosh laryngoscope varied and amounted to 85.9% and 83.6%, respectively (OR = 1.93; 95% CI: 0.82 to 4.51; I^2^ = 72%; *p* = 0.13; Figure 2). In turn, intubation with video tubes or scopes was associated with a statistically significantly higher first intubation success rate than intubation with DL (91.5% vs. 84.9%) (OR = 1.86; 95% CI: 1.16 to 2.99; I^2^ = 0%; *p* = 0.01). An inverse relationship was observed in the subgroup of channeled laryngoscopes: intubation with these devices compared with DL was associated with a lower first intubation success rate (82.9% vs. 89.0%) (OR = 0.61; 95% CI: 0.15 to 2.45; I^2^ = 47%; *p* = 0.49).

### 3.3. Overall Intubation Success Rate

Overall success rate was reported in 16 studies and equaled 99.8% for VL and 98.8% for DL (OR = 3.89; 95% CI: 0.95 to 15.93; I^2^ = 0; *p* = 0.06).

Subgroup analysis established a 100% overall intubation success rate for VL with a Macintosh shaped blade and for DL. Additionally, for channeled laryngoscopes and DL comparison, the overall success rate was 100% in both groups. On the other hand, the overall success rate with video tubes and scopes turned out to be 99.4% compared with 97.5% for DL (OR = 3.89; 95% CI: 0.95 to 15.93; I^2^ = 0%; *p* = 0.06).

### 3.4. Intubation Time

Mean intubation time for VL equaled 43.4 ± 30.4 s and was statistically significantly shorter than that with DL: 54.0 ± 56.3 s (MD = −11.87; 95% CI: −17.06 to −6.68; I^2^ = 99%; *p* < 0.001).

**Figure 3:**
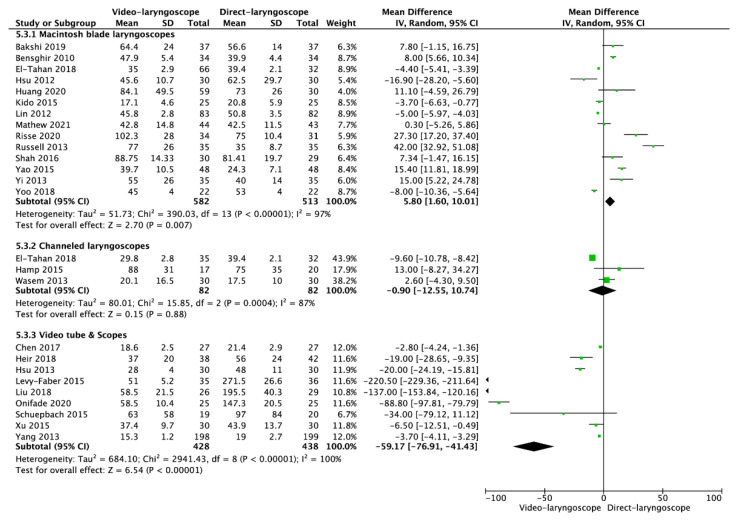
Forest plot of time to intubation in video-laryngoscope and direct-laryngoscope groups. The center of each square represents the weighted mean differences for individual trials, and the corresponding horizontal line stands for a 95% confidence interval. The diamonds represent pooled results. Legend: CI = confidence interval; MD = mean difference.

In the Macintosh blade laryngoscope subgroup, mean time of intubation amounted to 55.7 ± 30.4 s for VL and 48.6 ± 21.2 s for DL (MD = 5.80; 95% CI: 1.60 to 10.01; I^2^ = 97%; *p* = 0.007; Figure 3). In the channeled laryngoscope subgroup, intubation time was 38.3 ± 31.0 s for VL compared with 40.1 ± 28.5 s for DL (MD = −0.90; 95% CI: −12.55 to 10.74; I^2^ = 87%; *p* = 0.88). In the subgroup of video tubes and scopes, mean intubation time with VL equaled 28.3 ± 22.2 s and was statistically significantly shorter than that with DL: 65 ± 82.2 s (MD = −59.17; 95% CI: −76.91 to −41.43; I^2^ = 100%; *p* < 0.001).

### 3.5. Glottic View

Glottic view in accordance with Cormack–Lehane grade was reported in 15 studies. Good view, defined as Cormack–Lehane grade 1 or 2, varied in the VL and DL groups and equaled 93.1% and 88.1%, respectively (OR = 3.33; 95% CI: 1.18 to 9.41; I^2^ = 63%; *p* = 0.02).

In the Macintosh blade subgroup analysis, VL resulted in better visualization of the glottis than DL (96.5% vs. 87.8%) (OR = 5.40; 95% CI: 1.99 to 14.66; I^2^ = 45%; *p* < 0.001; Figures S3 and S4). The same relationship was observed in the channeled VL subgroup (75.6% vs. 63%, respectively) (OR = 4.78; 95% CI: 0.17 to 135.25; I^2^ = 80%; *p* = 0.36).

In the subgroup of video tubes and scopes, glottic visualization at Cormack–Lehane category grades 1–2 was observed in 92.9% for VL and 93.5% for DL (OR = 0.94; 95% CI: 0.30 to 2.99; I^2^ = 0%; *p* = 0.92).

### 3.6. External Laryngeal Manipulation

Ten studies reported ELM during the intubation process. Pooled analysis revealed that ELM was needed in 18.4% cases of VL compared with 42.8% in the DL group (OR = 0.28; 95% CI: 0.20 to 0.40; I^2^ = 69%; *p* < 0.001).

Subgroup analysis showed that ELM was less often required with VL than with DL in the Macintosh blade VL group (21.4% vs. 44.3%) (OR = 0.27; 95% CI: 0.12 to 0.60; I^2^ = 74%; *p* = 0.001), the channeled VL group (10.8% vs. 29.0%) (OR = 0.23; 95% CI: 0.03 to 1.97; I^2^ = 54%; *p* = 0.18), and the group of video tubes and scopes (0% vs. 46.7%) (OR = 0.02; 95% CI: 0.00 to 0.33; *p* = 0.007).

### 3.7. Adverse Events

Intubation with VL and DL exhibited a comparable rate of adverse events. A full list of the analyzed adverse events is presented in Table 2.

## 4. Discussion

The analysis of the success rate of the first intubation attempt, described in 26 studies (*n* = 2154), showed higher values in the VL group than for DL. However, there was no statistical significance, but only a trend toward significance (*p* = 0.07). These results contrasted with the data obtained by Liu et al., who investigated 1215 patients and confirmed a significant superiority of video devices (VL or video-stylet) over Macintosh laryngoscope (OR = 2.77; 95% CI: 1.92 to 4.00; *p* < 0.00001) [43]. The same authors showed no differences in subgroup analysis depending on the type of the video device used. In contrast, they maintained that intubation with video tubes or scopes was associated with a statistically significantly higher first attempt success rate than intubation with DL. An analysis of the total intubation success and failure rates revealed no significant differences between VL and DL (*p* = 006). However, the involved 17 randomized clinical trials exhibited low homogeneity values. Endotracheal intubation with DLTs, due to their specificity, is more difficult than that with standard equipment; therefore, it is important to maximize the probability of intubation, especially during the first attempt. For this purpose, the use of video tubes or scopes is recommended first.

One of the fundamental factors of perioperative safety is the duration of endotracheal intubation. Prolonged apnea time can lead to significant hypoxia with its consequences. Patients undergoing thoracic surgery are special cases. On one hand, they usually present with pulmonary diseases (e.g., chronic obstructive pulmonary disease, bronchial asthma, pneumoconiosis), which are initially associated with a high risk of hypoxemia and low oxygen reserve. As mentioned earlier, the use of DLT is a priori associated with prolonged intubation due to the device specificity. Our meta-analysis confirmed the superiority of VL over DL for significantly shorter DLT intubation times. It should be noted that differences in intubation time are unsure due to the very high heterogeneity of the data (I^2^ > 90%) and this problem also applies to other meta-analyses. Different data were provided by Liu et al., who demonstrated no significant difference in intubation time between any video device and the Macintosh laryngoscope [43]. This, as the authors themselves indicate, may have been influenced by a high level of heterogeneity when reporting intubation times across randomized controlled trials. In our study, as in the analysis by Kim et al., the use of video tubes or scopes was associated with significantly shorter intubation times [44].

The safety of endotracheal intubation and occurrence of adverse events are particularly influenced by the degree of laryngeal visualization and the need for external manipulation within the larynx. The use of VL allowed better glottic visualization (rated 1 and 2 on the Cormack–Lehane scale), and the need for ELM was described significantly less often. Subgroup analysis showed no need for ELM with video tubes or scopes.

DLT intubation involves several consecutive steps. First, the laryngeal entry is visualized; then, the bronchial tip is inserted behind the vocal cords; the tube is advanced toward the tracheal bifurcation; and, finally, the bronchial end of the tube is placed in the appropriate main bronchus [45]. The duration of this procedure is significantly longer than that of single-tube intubation and depends on many factors including visualization of the laryngeal entry. The technique is also associated with a higher risk of adverse events. Perioperative oral trauma with bleeding, damage to teeth and cheeks, distention, bronchospasm, and cardiac arrhythmias as well as postoperative sore throat and hoarseness are some of the complications related to endotracheal intubation [46,47]. During intubation with DLT, the risk of these complications is significantly higher, and esophageal intubation or tube displacement are particularly associated with this procedure [46]. In the material analyzed, DL was related to a higher risk of unintended esophageal intubation and, at the same time, a lower risk (but not statistically significant, *p* = 0.06) of tube misplacement. However, the homogeneity of the randomized controlled trials included in the study was low. The presented data are consistent with those provided by Liu et al., who confirmed that the use of VL was associated with a higher incidence of tube misplacement [43]. The occurrence of other adverse events such as oral bleeding, bronchospasm, sore throat, hoarseness, desaturation, cardiac arrhythmia, lip trauma, dental trauma, and cuff rupture was not associated with any of the intubation techniques used. These results, when compared with the available meta-analyses, are conflicting. In both studies by Liu et al. [43] and Kim et al. [44], the use of VL brought about a lower risk of intubation complications. Of these, the use of video-stylets was associated with a lower incidence of the described adverse events [44]. These discrepancies may be influenced by, among other factors, the lack of particular VL subgroup analysis in our study.

There are some limitations of our study that should be considered. These are due to several factors that affect the obtained results. The first limitation is the relatively small study group in most of the analyzed publications. Another problem is that in the majority of the publications, only patients with normal airways were included and studies were limited to a specific patient population. In some studies, intubation was performed by physicians experienced in endotracheal intubation. Another limitation is the use of specific types of DLTs. Moreover, it should be mentioned that due to the small number of studies using channeled-laryngoscopes, the conclusions on channeled-laryngoscopes were based on only 82 patients.

## 5. Conclusions

For double-lumen endotracheal intubation, VL offers shorter intubation time, better glottic view in accordance with Cormack–Lehane grade, and lower need for ELM, but comparable first intubation attempt success rate and overall intubation success rate compared with DL.

## Figures and Tables

**Table 1:** Summary characteristics of the included studies.

Study	Country	Study Design	Intubation Method	No. of Patients	Age	Sex, Male	BMI	ASA I or II
Bakshi et al., 2019	India	RCT	McGrath	37	46.9 (17)	25 (67.6%)	21.8 (3)	37 (100%)
			Macintosh	37	49.8 (16)	23 (62.2%)	23.0 (3)	37 (100%)
Bensghir et al., 2010	France	RCT	X-Lite	34	41.8 (9)	28 (82.3%)	24 (2.9)	34 (100%)
			Macintosh	34	44.6 (10)	29 (85.3%)	22.98 (2.19)	34 (100%)
Chen et al., 2017	China	RCT	Disposcope	27	59.5 (12.9)	9 (33.3%)	24.3 (3.0)	NS
			Macintosh	27	61.0 (11.4)	11 (40.7%)	23.8 (3.1)	NS
El-Tahan et al., 2018	Saudi Arabia	RCT	GlideScope	34	39.9 (17.52)	26 (76.5%)	NS	20 (58.8%)
			Airtraq	35	33.8 (13.37)	31 (88.6%)	NS	19 (54.3%)
			KingVision	32	31.3 (14.8)	27 (84.4%)	NS	20 (62.5%)
			Macintosh	32	27.5 (9.83)	19 (59.4%)	NS	22 (68.8%)
Hamp et al., 2015	Austria	RCT	Airtraq	17	56.8 (10.6)	9 (52.9%)	NS	16 (94.1%)
			Macintosh	20	63.4 (9.3)	11 (55.0%)	NS	18 (90.0%)
Heir et al., 2018	USA	RCT	ETView	38	NS	28 (73.7%)	29.8	NS
			Macintosh	42	NS	19 (45.2%)	27.0	NS
Hsu et al., 2012	Taiwan	RCT	GlideScope	30	40.1 (18.7)	7 (23.3%)	21.3 (3.4)	30 (100%)
			Macintosh	30	37.2 (15.4)	11 (36.7%)	23.0(5.6)	30 (100%)
Hsu et al., 2013	Taiwan	RCT	Trachway stylet	30	40 (15)	20 (66.7%)	21 (4)	NS
			Macintosh	30	47 (15)	22 (73.3%)	23 (4)	NS
Huang et al., 2020	China	RCT	GlideScope	29	58.45 (8.8)	11 (37.9%)	23.33 (3.29)	29 (100%)
			C-MAC	30	57.2 (9.6)	18 (60.0%)	22.82 (2.67)	30 (100%)
			Macintosh	30	54.57 (11.78)	20 (66.7%)	24.32 (3.78)	30 (100%)
Kido et al., 2015	Japan	RCT	McGrath	25	66.6 (11.3)	15 (60.0%)	22.3 (3.2)	13 (52.0%)
			Macintosh	25	67.9 (15.0)	16 (64.0%)	21.9 (4.6)	11 (44.0%)
Levy-Faber et al., 2015	Izrael	RCT	VivaSight	35	68 (61–74)	21 (60.0%)	NS	19 (54.3%)
			Macintosh	36	67 (61–75)	18 (50.0%)	NS	20 (55.6%)
Lin et al., 2012	China	RCT	CEL-100	83	58.2 (9.6)	55 (66.3%)	22.9 (2.7)	76 (91.6%)
			Macintosh	82	57.6 (9.4)	52 (63.4%)	23.1 (2.8)	76 (92.7%)
Liu et al., 2018	China	RCT	VivaSight	26	39.5 (13.5)	16 (61.5%)	22.9 (3.1)	NS
			Macintosh	29	40.0 (13.3)	18 (62.1%)	23.6 (3.6)	NS
Maharaj et al., 2006	Ireland	RCT	AirTraq	30	43.8 (16.8)	11 (36.7%)	27.1 (6.1)	NS
			Macintosh	30	41.1 (16.9)	11 (36.7%)	27.7 (5.7)	NS
Mathew et al., 2021	India	RCT	C-MAC	44	36.3	29 (65.9%)	22.1	42 (95.5%)
			Macintosh	43	40.4	28 (65.1%)	22.5	39 (90.7%)
Onifade et al., 2020	USA	RCT	VivaSight	25	55.3 (6.6)	11 (44.0%)	27.5 (2.8)	4 (16.0%)
			Macintosh	25	53.5 (5.2)	13 (52.0%)	28.7 (1.6)	4 (16.0%)
Risse et al., 2020	Germany	RCT	GlideScope	34	66.3 (4.9)	25 (73.5%)	25.9 (1.4)	10 (29.4%)
			Macintosh	31	59.3 (3.8)	25 (80.6%)	26.6 (1.9)	10 (32.3%)
Russell et al., 2013	Canada	RCT	GlideScope	35	59 (12)	15 (42.9%)	26 (5)	8 (22.9%)
			Macintosh	35	62 (14)	18 (51.4%)	26 (4)	5 (14.3%)
Schuepbach et al., 2015	Switzerland	RCT	VivaSight	19	57 (17)	9 (47.4%)	23 (4)	11 (57.9%)
			Macintosh	20	63 (10)	10 (50.0%)	24 (3)	8 (40.0%)
Shah et al., 2016	India	RCT	C-MAC	30	54.57 (11.06)	22 (73.3%)	NS	30 (100%)
			Macintosh	30	52.13 (12.69)	20 (66.7%)	NS	30 (100%)
Wasem et al., 2013	Germany	RCT	AirTraq	30	63 (10)	22 (73.3%)	27.4 (2.8)	16 (53.3%)
			Macintosh	30	55 (19)	19 (63.3%)	27.1 (6.2)	17 (56.7%)
Xu et al., 2015	China	RCT	SOS stylet	30	50.1 (11.1)	14 (46.7%)	23.4 (3.0)	26 (86.7%)
			Macintosh	30	46.3 (16.1)	17 (56.7%)	24.0 (4.9)	27 (90.0%)
Yang et al., 2013	Republic of Korea	RCT	Optiscope	198	55.5 (9.7)	140 (70.7%)	23.2 (2.9)	NS
			Macintosh	199	56.5 (9.0)	150 (75.4%)	23.4 (3.1)	NS
Yao et al., 2015	China	RCT	McGrath	48	47.6 (13.8)	33 (68.8%)	22.0 (3.4)	47 (97.9%)
			Macintosh	48	47.8 (16.3)	33 (68.8%)	21.9 (3.0)	44 (91.7%)
Yi et al., 2013	China	RCT	GlideScope	35	NS	NS	NS	NS
			Macintosh	35	NS	NS	NS	NS
Yao et al., 2018	Korea	RCT	McGrath	22	47.5 (2.9)	14 (63.6%)	NS	22 (100%)
			Macintosh	22	49.3 (2.6)	14 (63.6%)	NS	22 (100%)

Legend: NS: Not specified; RCT: Randomized controlled trial.

**Table 2:** Pooled analysis of adverse events reported in the included trials.

Type of Adverse Event	No. of Studies	Events/Participants		Events		Heterogeneity between Trials		*p*-Value for Differences across Groups
		VL	DL	RR	95% CI	*p*-Value	I^2^ Statistic	
Oral bleeding	10	35/400 (8.8%)	52/373 (13.9%)	0.67	0.37 to 1.20	0.14	34%	0.18
Blood on laryngoscope blade	5	10/180 (5.6%)	10/176 (5.7%)	0.93	0.33 to 2.65	0.29	19%	0.90
Bronchospasm	5	0/162 (0.0%)	4/163 (2.5%)	0.19	0.02 to 1.60	0.97	0%	0.13
Sore throat	15	149/640 (23.3%)	174/549 (31.7%)	0.86	0.67 to 1.09	<0.001	79%	0.22
Hoarseness	13	118/554 (21.3%)	140/459 (30.5%)	0.80	0.57 to 1.13	<0.001	83%	0.21
Desaturation	6	10/246 (4.1%)	24/241 (10.0%)	0.48	0.18 to 1.26	0.22	30%	0.13
Cardiac arrhythmia	3	6/98 (6.1%)	12/95 (12.6%)	0.52	0.17 to 1.56	0.28	16%	0.24
Lip trauma	3	4/170 (2.4%)	1/98 (10.2%)	2.07	0.34 to 12.76	0.57	0%	0.43
Dental trauma	6	0/290 (0.0%)	0/193 (0.0%)	NE	NE	NA	NA	NA
Esophageal intubation	4	0/187 (0.0%)	7/185 (3.8%)	0.13	0.02 to 0.98	0.90	0%	0.05
Cuff rupture	5	14/288 (4.9%)	14/217 (6.5%)	0.84	0.23 to 3.12	0.11	51%	0.80
Tube misplacement	5	30/190 (15.8%)	10/162 (6.2%)	1.99	0.94 to 4.19	0.35	8%	0.07

Legend: VL: video-laryngoscopy; DL: direct laryngoscopy; NE: not estimable; NA: not applicable; RR: risk ratio.

## Data Availability

Not applicable.

## Supplementary Materials

The following are available online at https://www.mdpi.com/article/10.3390/jcm10235524/s1, Table S1: Methodology characteristics among the included trials, Table S2: PRISMA Checklist, Table S3: Polled analysis of patient characteristics, Figure S1: Distribution of American Society of Anesthesiologists grades among 1305 patients, Figure S2: Distribution of Mallampati class among 1795 patients, Figure S3: Forest plot of Cormack–Lehane grades 1 or 2 in video-laryngoscope and direct-laryngoscope groups, Figure S4: Distribution of glottis visualization in Cormack–Lehane grades among 1500 patients, Figure S5: A summary table of the review authors’ judgements for each risk of bias item for each randomized study, Figure S6: A plot of the distribution of the review authors’ judgements across randomized studies for each risk of bias item.
