# Supplementary material for: Efficacy and Safety of Video-Laryngoscopy versus Direct Laryngoscopy for Double-Lumen Endotracheal Intubation: A Systematic Review and Meta-Analysis
Source: J Clin Med. 2021 Nov 25;10(23):5524. doi: 10.3390/jcm10235524 (PMC8658072; doi:10.3390/jcm10235524)
Supplement: Supplementary file 1 [file jcm-10-05524-s001.zip › jcm-1458232-supplementary.pdf]

Efficacy and safety of video-laryngoscopy vs. direct laryngoscopy for double-lumen endotracheal intubation: a systematic review and meta-analysis

Supplementary Digital File

Content:

Table S1. Methodology characteristics among included trials. 2

Table S2. PRISMA Checklist....8

Table S3. Polled analysis of patients characteristics....11

Figure S1. Distribution of American Society of Anesthesiologists grades among 1,305 patients. 1

Figure S2. Distribution of Mallampati class among 1,795patients. 1

Figure S3. Forest plot of Cormack-Lehane 1 or 2 grade in video-laryngoscope and direct-laryngoscope groups 2

Figure S4. Distribution of glottis visualization in Cormack-Lehane grade among 1,500 patients 3

Figure S5. A summary table of review authors' judgements for each risk of bias item for each randomized study. 4

Figure S6. A plot of the distribution of review authors' judgements across randomized studies for each risk of bias item. 5

Table S1. Methodology characteristics among included trials.

Study	Inclusion criteria	Exclusion criteria	Primary outcome(s)	Findings
Bakshi et al. 2019	Patients with American Society of Anesthesiologist physical grading I–II, posted for elective surgery needing lung isolation.	History of or anticipated difficult airway on clinical examination [including Mallampati Class (MPC) III and IV, thyromental distance less than 6.5 cm, sternomental distance less than 12.5 cm, interincisor gap less than 3 cm, body mass index (BMI) >30 kg/m2] and presence of indications for rapid sequence induction of anaesthesia	The time required for intubation.	TTI for DLT insertion was similar with VL and DL. However, VL was associated with better glottis visualization, reduced need of external laryngeal manipulation and fewer complications.
Bensghir et al. 2010	Patients American Society of Anesthesiologists (ASA) physical status I and II	Criteria indicating possible difficult intubation.	The time required for intubation.	The use of a video laryngoscope reduces the time required for intubation with a DLT compared with the direct laryngoscopy in elective thoracic surgery
Chen et al. 2017	Male or female ASA physical status 1–3 patients who were between 20 and 80 years of age, and who required double-lumen tubes to maintain pulmonary isolation during thoracic surgery.	Emergency surgery; known history of difficult intubation or possible intubation difficulties (Mallampati class > 3, mouth opening < 2 cm, thyromental distance < 6 cm); body mass index (BMI) > 35 kg.m 2; unstable vital signs or requiring the use of vasopressors or inotropic agents; history of head, neck or throat surgery; and those who did not provide written informed consent. In addition, patients in whom tracheal intubation was judged to be difficult at the time of surgery were also not studied.	The success rate at the first placement attempt.	The Disposcope increased the success rate of double-lumen tube placement, and shortened the total operation time when compared with standard placement with confirmation using fiberoptic bronchoscopy, and may replace the conventional method.
El-Tahan et al. 2018	Patients between 18 and 70 years old with an American Society of Anesthesiologists physical status classification of II to III who were undergoing general anesthesia with DLT intubation for one-lung ventilation during elective thoracic surgery.	Patients with a New York Heart Association functional classification of III to IV, a forced expiratory volume in 1 second or forced vital capacity of less than 50% of predicted values, severe asthma, pregnancy, or an anticipated or known difficult intubation (eg, because of a body mass index 440 kg/m^2 or incisor gap o3 cm). Patients also were excluded if a rapid sequence induction was indicated, such as those with risk factors for aspiration of gastric contents, or if	The time required for achieving successful DLT intubation (defined as the time from the laryngoscope passing the central incisors to the tip of the DLT passing beyond the glottis), as confirmed visually by the anesthesiologist in the Macintosh group or by the investigator using the display screen in	When used by operators with mixed experience, the channeled Airtraq required less time for DLT intubation and was easier to use than the GlideScope, although failures did occur with the Airtraq, whereas they did not occur with the other systems.

		postoperative ventilation was planned.	the VL groups, regardless of the number of laryngoscopy attempts	
Hamp et al. 2015	Adult patients of ASA class 1-3 undergoing elective surgery with the need for DLT tube placement.	Patients taking medication with anti-hyper- tensive or beta-blocking agents on the day of surgery; cardiac arrhythmias and history of previous difficult intubation procedures, as these may inappropriately prolong intubation at- tempts or require awake fiberoptic intubation.	Arterial blood pressure, heart rate, catecholamine levels, bispectral index and duration of the intubation procedure.	The use of the Double-lumen Airtraq® laryngoscope provides no benefit regarding stress response compared to the MacIntosh laryngoscope.
Heir et al. 2018	Patients who needed one-lung ventilation for their proposed surgery, patients had to be older than 18 years of age, and prior to using the VDLT all anesthesiologists had to either observe or personally perform 3 intubations with the VDLT.	Patients with known tracheobronchial anatomic anomalies, those going for emergency procedures, those with known difficult airways, surgeries in which other lung isolation devices or techniques may be warranted (tracheostomy, nasal intubation), patients requiring endotracheal tube sizes not available in DLT or VDLT, and patients requiring a rightsided DLT or VDLT.	To estimate the rate of FOB use with the VDLT during thoracic surgeries and to compare this with the rate of FOB use with the conventional DLT.	This study demonstrated a reduction of 86.8% in FOB use, which was a similar reduction found in other published studies.
Hsu et al. 2012	Adult patients of ASA physical status 1–2, requiring a DLT for thoracic surgery.	Risk of regurgitation and pulmonary aspiration, history of gastro-oesophageal reflux, pregnancy, scheduled tracheostomy and planned postoperative ventilation in ICU. In addition, patients with a potentially difficult laryngoscopy as suggested by limited neck extension (< 35°), a distance between the tip of the patient's mandible and the thyroid notch < 7 cm, or a sternomental distance < 12.5 cm with the head fully extended and the mouth closed.	Intubation success rates and the time needed to intubate.	Double-lumen tube intubation in patients with predicted normal laryngoscopy is easier using the GlideScope video-laryngoscope than the Macintosh laryngoscope.
Hsu et al. 2013	Patients categorized as ASA physical status 1 to 3, older than 18 years, who required endobronchial intubation with a double-lumen tube for thoracic surgery.	Patients with an increased risk or history of gastro-esophageal reflux, or who were pregnant, scheduled for tracheostomy, or were expected to require prolonged postoperative ventilation.	The time required for intubation.	Compared with direct laryngoscopy, the Trachway video stylet facilitated faster endobronchial intubation with a left-sided double-lumen tube and decreased hoarseness on the first postoperative day in patients with normal airways.

Huang et al. 2020	Age 18–75 years old; ASA I–II, BMI < 35 kg/m², with Mallampati score of 1 or 2. All Mallampati scores were assigned by the same observer.	Presence of any predictors of difficult intubation; Mallampati score > =3; inter-incisor distance <3cm; thyromental distance < 6 cm; neck extension < 80°from neck flexion; cervical spine instability; history of difficult endotracheal intub- ation or difficult mask ventilation; and severe pulmonary ventilation dysfunction or risk of pulmonary aspiration.	The time required for intubation.	Compared with the Macintosh laryngoscope, the GlideScope® and C-MAC®(D) video-laryngoscopes may not be recommended as the first choice for routine DLT intubation in patients with predicted normal airways.
Kido et al. 2015	Patients aged 20 to 85 years who were to undergo general anesthesia with 1-lung ventilation.	Any contraindication for rapid induction (such as full stomach, gastroesophageal reflux), patients with suspected invasion of cancer in the trachea, or anticipated difficult airway patients (such as difficult head tilting, mouth opening).	The time required for intubation.	The McGrath demonstrated a better intubation profile compared with the Macintosh laryngoscope, possibly due to its ease of use for double-lumen endotracheal tube intubation.
Levy-Faber et al. 2015	Adult patients who underwent elective video-assisted thoracoscopic surgery for lung lobectomy.	A requirement for rapid sequence induction, known tracheal pathology and anticipated difficult intubation.	The time required for intubation.	The VivaSight DL enables significantly more rapid intubation compared with the conventional double-lumen tube.
Lin et al. 2012	Adults who were scheduled for elective open thoracic surgery requiring double-lumen tube insertion for one-lung ventilation.	Limited mouth opening, ASA physical status of 4 > 3, age < 18 years or a history of known difficult airway. All patients under- went a pre-operative airway assessment and the Mallampati score, inter-incisor gap and thyromental distance.	The time required for intubation.	The CEL-100 video-laryngoscope is superior to the Macintosh laryngoscope blade for double-lumen tube insertion.
Liu et al. 2018	Adult patients who underwent elective VATS for lung lobectomy.	American Society of Anesthesiologists class 4 or higher, age less than 18 years, risk of gastric aspiration, abnormalities of the upper airway, polyps requiring rapid sequence induction, known tracheal pathology, and anticipated difficult intubation.	To demonstrate that by using the ETView system, correct positioning of the DLT and the endobronchial blocker could be achieved without a FOB in patients undergoing left lung surgery.	The ETView tube to be helpful in the endotracheal intubation and continuous surveillance of tube position in patients with video-assisted thoracoscopic lobectomy. The ETView single lumen endotracheal tube had fewer associated complications and is superior to the 2 double-lumen tubes.
Maharaj et al. 2006	ASA physical status I–III patients, aged 18 years of age or older, scheduled for surgical procedures requiring tracheal intubation.	Risk factors for gastric aspiration and/or risk factors for difficult intubation (Mallampatti class III or IV; thyromental distance less than 6 cm; interincisor distance less than 4.0 cm) were present or	The time required for intubation and the intubation difficulty scale (IDS) score.	The Airtraq laryngoscope offers a new approach for the management of the normal airway. The Airtraq reduced the difficulty of tracheal intubation and the degree of hemodynamic

		where there was a history of relevant drug allergy.		stimulation compared to the Macintosh laryngoscope in patients at low risk for difficult laryngoscopy and intubation, in this first randomized clinical trial with this device.
Mathew et al. 2021	Patients between the ages of 18 to 70 years.	Pregnant patients, patients with anticipated difficult airway – limited neck extension, thyromental distance less than 6.5 cm, height <150 cm, BMI greater than 30, Mallampatti 4, ASA IV patients, and patients at risk of aspiration. Patients in whom there was a failure to intubate after three attempts were excluded from the study.	To compare the mean time taken for DLT intubation with CMAC (Mac 3) and Macintosh laryngoscope blade and the secondary objectives included the hemodynamic response to intubation, the level of difficulty using the intubation difficulty scale (IDS), and complications associated with intubation.	Macintosh blade is as good as CMAC (mac 3) blade to facilitate DLT intubation in adult patients with no anticipated airway difficulty, however CMAC was superior as it offers better laryngoscopic view, needed less force, and fewer external laryngeal manipulations.
Onifade et al. 2020	18–90 years old, scheduled for a thoracic surgery requiring single lung ventilation, and not emergent.	Pregnant or nursing women, patients with known or suspected difficult airway, and patients with a contraindication for left-sided DLT insertion (e.g., left-sided bronchial mass).	Rate of FOB use.	This study demonstrated a significantly lower rate of FOB use when using a VS-DLT compared to a c-DLT. Placement of the VS-DLT was significantly quicker and malposition during surgery occurred significantly less than with the c-DLT. While intubating with a VS-DLT provides clinical benefits, it may not result in significant cost reductions when compared to a c-DLT.
Risse et al. 2020	Adult patients scheduled for elective thoracic surgery requiring general anesthesia with the need of a DLT for lung separation with American Society of Anesthesiologists physical status I–IV.	Patient age < 18 years, non-elective surgery, pregnancy, scheduled rapid-sequence induction, contraindication for DLT insertion; contraindication to one-lung ventilation as well as abnormal physical status of the Cervical spine (e.g., after C-spine trauma, Bechterew's disease).	The time required for intubation.	Video-laryngoscopy using the GlideScope®-Titanium shortly prolongs DLT intubation duration compared to direct laryngoscopy but improves the view. Objective intubation trauma but not subjective complaints are reduced.
Russell et al. 2013	Patients aged over 18 years undergoing elective surgical procedures requiring endobronchial intubation with a left-sided DLT.	A history of previous failed or difficult tracheal intubation or if difficult tracheal intubation was anticipated (two risk factors of Mallampati score three or	The time required for intubation.	The GlideScope more difficult to use compared with the Macintosh laryngoscope for endobronchial intubation

		greater, incisor gap < 3.5 cm, thyromental distance < 6.5 cm, and reduced neck extension and flexion). Other exclusion criteria were the following: alternative method of tracheal intubation indicated (e.g. rapid sequence intubation); contraindication to a left DLT; contraindication to one-lung ventilation; anticipated difficult bag-mask ventilation of the lungs; and body mass index > 40 kg.m[2].		with a DLT, and we cannot therefore recommend its routine use in thoracic anesthesia in patients with an anticipated normal airway.
Schuepbach et al. 2015	Adults who were to undergo elective thoracic surgery that required single-lung ventilation.	Patients more than 90 yr of age and those with American Society of Anesthesiologists physical status IV or V, a body mass index [45 kg/m[2], and/or any contraindications to use of a left-sided 37-Fr double-lumen tube. Patients who had had thoracic surgery within the last four weeks, a systemic infection or suspected tuberculosis, or had been previously diagnosed with or suspected of having a difficult airway were also excluded.	The time required for intubation.	The VivaSight DLT camera allowed faster insertion and facilitated initial positioning. It also confirmed proper tube positioning intraoperatively and facilitated repositioning when necessary.
Shah et al. 2016	Age 18–80 years, American Society of Anesthesiologists (ASA) physical status 1–2, suffering from malignancy and requiring a DLT for elective thoracic surgery (oesophagectomy, pulmonary metastasectomy, lobectomy and pneumonectomy).	Risk of regurgitation and pulmonary aspiration, patients with tracheobronchial masses or compression, patients with <70% predicted forced expiratory volume in 1s, <80% predicted forced vital capacity, a PaO_2 <9.3 kPa while breathing air and mouth opening <1.5 cm.	The time required for intubation.	D-blade video-laryngoscope is a useful alternative to the standard Macintosh laryngoscope for routine DLT insertion.
Wasem et al. 2013	Patients over 18 years of age and American Society of Anesthesiologists' physical status class I–III required one-lung anesthesia due to the surgical procedure.	Pregnancy or refusal by the patient.	The time required for intubation.	There was no significant difference between the Airtraq and the Macintosh laryngoscopes regarding the time needed to insert a double-lumen tube during elective thoracic surgery. Only subtle enhancement of visualisation and a higher incidence of hoarseness were observed in the Airtraq group. The Airtraq device did not result in superior patient safety in this setting.
Xu et al. 2015	Patients undergoing elective thoracic surgery.	Not meet inclusion criteria.	The time required for intubation.	By comparison of the Macintosh laryngoscope, the SPS provides faster DLT intubation and

				causes less oral Mucosal or dental injury.
Yang et al. 2013	Patients aged18–80yr with ASA I–III who required DLT insertion for thoracic surgery.	Patients with increased risk of pulmonary aspiration, planned tracheostomy, or a requirement for rapid sequence induction	Success rate, intubation time, number of attempts at intubation, vocal cord view during intubation, need for external manipulation, and the incidences of oral mucosal or dental injury.	The OptiScope provides faster tracheal intubation and a higher success rate for the first intubation with less trauma and a better vocal cord view than the Macintosh laryngoscope.
Yao et al. 2015	Adult patients between 18–70 years old, of ASA physical status 1–3, scheduled for thoracic surgery requiring intubation with a double-lumen tube for one-lung ventilation.	Patients with a simplified airway risk index score ≥4. Other exclusion criteria included an increased risk of pulmonary aspiration and planned tracheostomy.	The time required for intubation.	In patients with a low airway risk index score requiring intubation with a double-lumen tracheal tube, the Macintosh laryngoscope is used as the first device and the McGrath video-laryngoscope is used only if this provides a poor glottic view.
Yi et al. 2013	ASA Ⅰ-Ⅲ patients, aged 18-75 yr,scheduled for thoracic surgery and requiring one-lung ventilation	Pregnancy or refusal by the patient.	The time required for intubation.	GlideScope video-laryngoscope can provide a better exposure of glottis and improvement in the intubating conditions,but the method is more complex and the response to intubation is stronger than Macintosh laryngoscope for DLT intubation.
Yao et al. 2018	Patients, 19 to 60 years of age, scheduled for thoracic surgery and requiring one-lung ventilation.	Patients required rapid-sequence intubation, had a history of difficult intubation, cervical spine instability or cervical myelopathy, or a tendency to bleed.	The time required for intubation.	The McGrath video-laryngoscope improved glottic view and resulted in lower overall intubation difficulty scale score in patients with in-line stabilization.

Legend: ASA: American Society of Anesthesiologist physical grading; BMI: body mass index; DLT: double-lumen tube;.

Table S2. PRISMA Checklist.

Section and Topic	Item #	Checklist item	Location where item is reported
		**TITLE**	
Title	1	Identify the report as a systematic review.	1
		**ABSTRACT**	
Abstract	2	See the PRISMA 2020 for Abstracts checklist.	1
		**INTRODUCTION**	
Rationale	3	Describe the rationale for the review in the context of existing knowledge.	1,2
Objectives	4	Provide an explicit statement of the objective(s) or question(s) the review addresses.	2
		**METHODS**	
Eligibility criteria	5	Specify the inclusion and exclusion criteria for the review and how studies were grouped for the syntheses.	2
Information sources	6	Specify all databases, registers, websites, organisations, reference lists and other sources searched or consulted to identify studies. Specify the date when each source was last searched or consulted.	2
Search strategy	7	Present the full search strategies for all databases, registers and websites, including any filters and limits used.	2
Selection process	8	Specify the methods used to decide whether a study met the inclusion criteria of the review, including how many reviewers screened each record and each report retrieved, whether they worked independently, and if applicable, details of automation tools used in the process.	2
Data collection process	9	Specify the methods used to collect data from reports, including how many reviewers collected data from each report, whether they worked independently, any processes for obtaining or confirming data from study investigators, and if applicable, details of automation tools used in the process.	2
Data items	10a	List and define all outcomes for which data were sought. Specify whether all results that were compatible with each outcome domain in each study were sought (e.g. for all measures, time points, analyses), and if not, the methods used to decide which results to collect.	2

	10b	List and define all other variables for which data were sought (e.g. participant and intervention characteristics, funding sources). Describe any assumptions made about any missing or unclear information.	2
Study risk of bias assessment	11	Specify the methods used to assess risk of bias in the included studies, including details of the tool(s) used, how many reviewers assessed each study and whether they worked independently, and if applicable, details of automation tools used in the process.	2
Effect measures	12	Specify for each outcome the effect measure(s) (e.g. risk ratio, mean difference) used in the synthesis or presentation of results.	3
Synthesis methods	13a	Describe the processes used to decide which studies were eligible for each synthesis (e.g. tabulating the study intervention characteristics and comparing against the planned groups for each synthesis (item #5)).	3
	13b	Describe any methods required to prepare the data for presentation or synthesis, such as handling of missing summary statistics, or data conversions.	3
	13c	Describe any methods used to tabulate or visually display results of individual studies and syntheses.	3
	13d	Describe any methods used to synthesize results and provide a rationale for the choice(s). If meta-analysis was performed, describe the model(s), method(s) to identify the presence and extent of statistical heterogeneity, and software package(s) used.	3
	13e	Describe any methods used to explore possible causes of heterogeneity among study results (e.g. subgroup analysis, meta-regression).	3
	13f	Describe any sensitivity analyses conducted to assess robustness of the synthesized results.	3
Reporting bias assessment	14	Describe any methods used to assess risk of bias due to missing results in a synthesis (arising from reporting biases).	3
Certainty assessment	15	Describe any methods used to assess certainty (or confidence) in the body of evidence for an outcome.	3
		**RESULTS**	
Study selection	16a	Describe the results of the search and selection process, from the number of records identified in the search to the number of studies included in the review, ideally using a flow diagram.	3
	16b	Cite studies that might appear to meet the inclusion criteria, but which were excluded, and explain why they were excluded.	3
Study characteristics	17	Cite each included study and present its characteristics.	3
Risk of bias in studies	18	Present assessments of risk of bias for each included study.	3

Results of individual studies	19	For all outcomes, present, for each study: (a) summary statistics for each group (where appropriate) and (b) an effect estimate and its precision (e.g. confidence/credible interval), ideally using structured tables or plots.	5-7
	20a	For each synthesis, briefly summarise the characteristics and risk of bias among contributing studies.	5-7
Results of syntheses	20b	Present results of all statistical syntheses conducted. If meta-analysis was done, present for each the summary estimate and its precision (e.g. confidence/credible interval) and measures of statistical heterogeneity. If comparing groups, describe the direction of the effect.	5-7
	20c	Present results of all investigations of possible causes of heterogeneity among study results.	5-7
	20d	Present results of all sensitivity analyses conducted to assess the robustness of the synthesized results.	5-7
Reporting biases	21	Present assessments of risk of bias due to missing results (arising from reporting biases) for each synthesis assessed.	5-7
Certainty of evidence	22	Present assessments of certainty (or confidence) in the body of evidence for each outcome assessed.	5-7
**DISCUSSION**			
Discussion	23a	Provide a general interpretation of the results in the context of other evidence.	8,9
	23b	Discuss any limitations of the evidence included in the review.	8,9
	23c	Discuss any limitations of the review processes used.	9
	23d	Discuss implications of the results for practice, policy, and future research.	9
**OTHER INFORMATION**			
Registration and protocol	24a	Provide registration information for the review, including register name and registration number, or state that the review was not registered.	2
	24b	Indicate where the review protocol can be accessed, or state that a protocol was not prepared.	2
	24c	Describe and explain any amendments to information provided at registration or in the protocol.	10
Support	25	Describe sources of financial or non-financial support for the review, and the role of the funders or sponsors in the review.	10
Competing interests	26	Declare any competing interests of review authors.	10
Availability of data, code and other materials	27	Report which of the following are publicly available and where they can be found: template data collection forms; data extracted from included studies; data used for all analyses; analytic code; any other materials used in the review.	10

Table S3. Polled analysis of patients characteristics.

Parameter	No. of studies	Value		Events		Heterogeneity between trials		P-value for differences across groups
		VL	DL	OR or MD	95% CI	P-value	I^2 statistic	
Age, years	22	52.5 ± 14.8	53.4 ± 14.5	0.77	-0.82 to 2.36	<0.001	69%	0.34
Sex male	24	670/1,058 (63.3%)	602/967 (62.3%)	1.01	0.84 to 1.23	0.43	2%	0.89
Weigh, kg	17	65.6 ± 13.0	66.3 ± 13.7	-2.10	-3.83 to -0.38	<0.001	64%	0.02
Heigh, cm	16	165.6 ± 8.2	165.4 ± 9.5	-0.65	-1.93 to 0.64	<0.001	72%	0.32
BMI	17	23.5 ± 3.4	23.8 ± 3.8	-0.39	-0.71 to -0.07	0.39	6%	0.02
Mouth opening, cm	10	4.5 ± 1.0	4.5 ± 0.9	0.03	-0.10 to 0.16	0.11	37%	0.64
Thyromental distance, cm	10	7.2 ± 1.5	7.5 ± 1.4	-0.02	-0.23 to 0.18	0.001	68%	0.83

Legend: BMI = body mass index; CI = confidence interval; DL = direct laryngoscope; MD = mead difference;. OR = odds ratio; VL = video-laryngoscope.

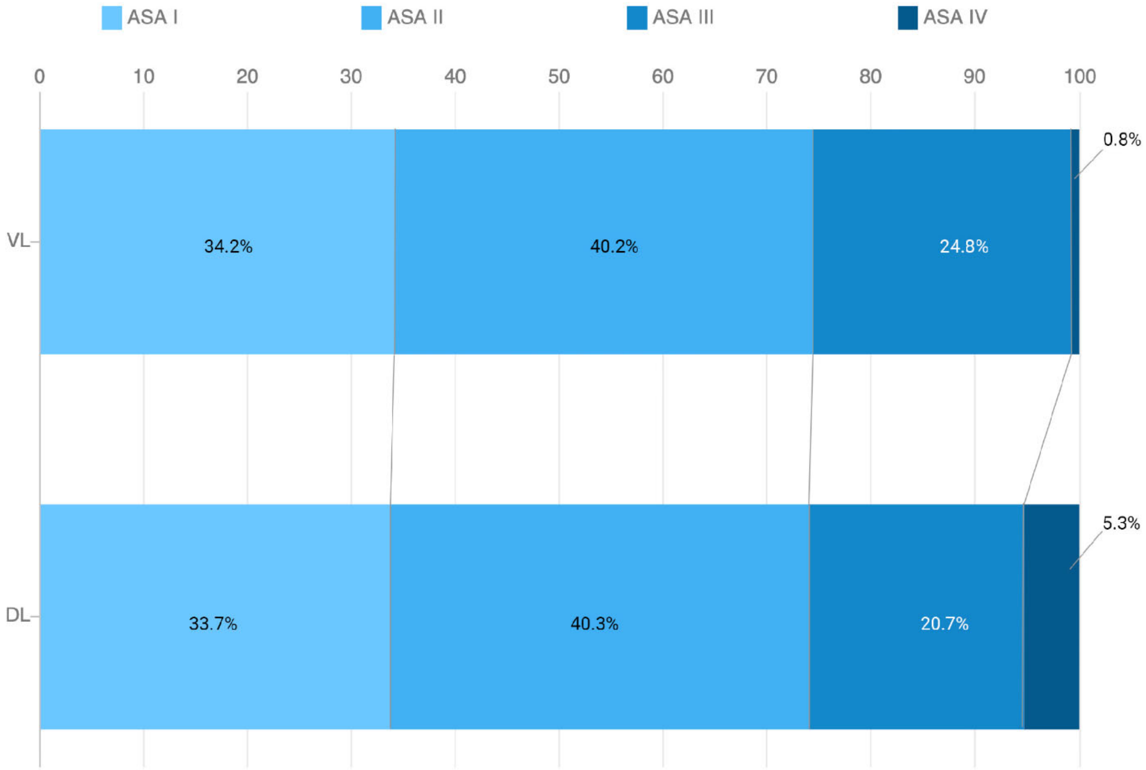

Figure S1. Distribution of American Society of Anesthesiologists grades among 1,305 patients.

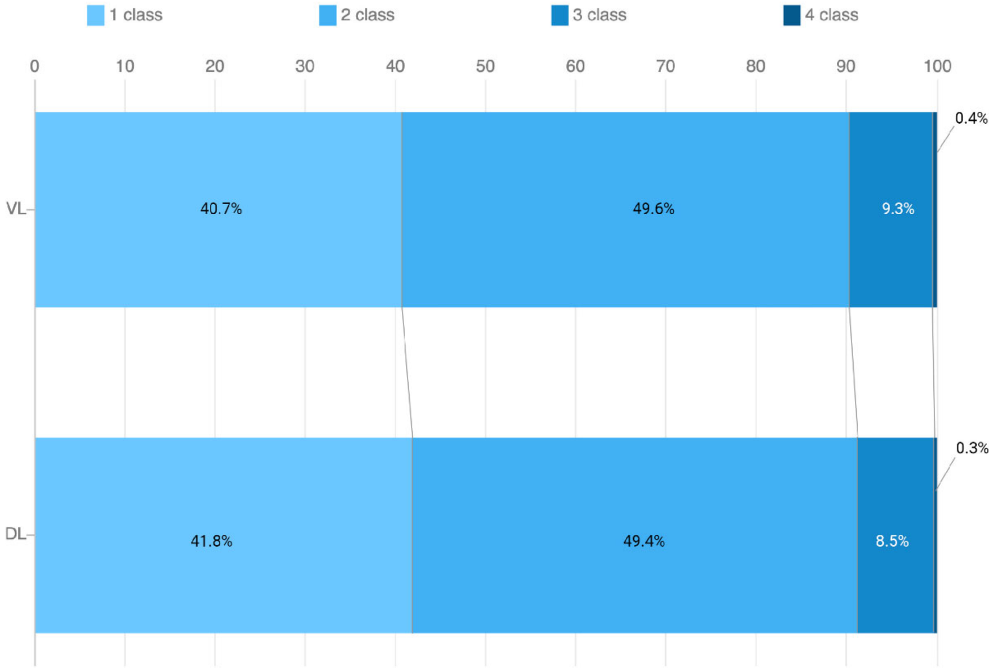

Figure S2. Distribution of Mallampati class among 1,795patients.

Study or Subgroup	VL Events	VL Total	DL Events	DL Total	Weight	Odds Ratio M-H, Random, 95% CI
**5.4.1 Macintosh blade laryngoscopes**						
Bakshi 2019	37	37	36	36		Not estimable
Bensghir 2010	32	34	24	34	15.6%	6.67 [1.34, 33.28]
El-Tahan 2018	54	66	28	32	18.9%	0.64 [0.19, 2.18]
Huang 2020	59	59	23	30	8.2%	37.98 [2.08, 691.80]
Kido 2015	25	25	20	25	8.0%	13.68 [0.71, 262.17]
Lin 2012	82	83	77	82	11.7%	5.32 [0.61, 46.61]
Purugganan 2012	45	46	33	40	11.9%	9.55 [1.12, 81.37]
Risse 2020	34	34	30	31	7.0%	3.39 [0.13, 86.43]
Shah 2016	30	30	28	29	7.0%	3.21 [0.13, 82.07]
Yao 2015	48	48	48	48		Not estimable
Yoo 2018	21	22	12	22	11.7%	17.50 [1.99, 153.97]
**Subtotal (95% CI)**		**484**		**409**	**100.0%**	**5.40 [1.99, 14.66]**
Total events	467		359			
Heterogeneity: $Tau^2 = 0.99$; $Chi^2 = 14.66$, df = 8 (P = 0.07); $I^2 = 45\%$						
Test for overall effect: Z = 3.31 (P = 0.0009)						
**5.4.2 Channeled laryngoscopes**						
El-Tahan 2018	15	35	12	32	58.1%	1.25 [0.47, 3.33]
Hamp 2015	17	17	16	30	41.9%	30.76 [1.70, 558.05]
Wasem 2013	30	30	30	30		Not estimable
**Subtotal (95% CI)**		**82**		**92**	**100.0%**	**4.78 [0.17, 135.25]**
Total events	62		58			
Heterogeneity: $Tau^2 = 4.75$; $Chi^2 = 4.90$, df = 1 (P = 0.03); $I^2 = 80\%$						
Test for overall effect: Z = 0.92 (P = 0.36)						
**5.4.3 Video tube & Scopes**						
Heir 2018	36	38	38	42	43.1%	1.89 [0.33, 10.99]
Levy-Faber 2015	32	35	35	36	24.9%	0.30 [0.03, 3.08]
Liu 2018	24	26	27	29	32.1%	0.89 [0.12, 6.81]
**Subtotal (95% CI)**		**99**		**107**	**100.0%**	**0.94 [0.30, 2.99]**
Total events	92		100			
Heterogeneity: $Tau^2 = 0.00$; $Chi^2 = 1.53$, df = 2 (P = 0.47); $I^2 = 0\%$						
Test for overall effect: Z = 0.10 (P = 0.92)						

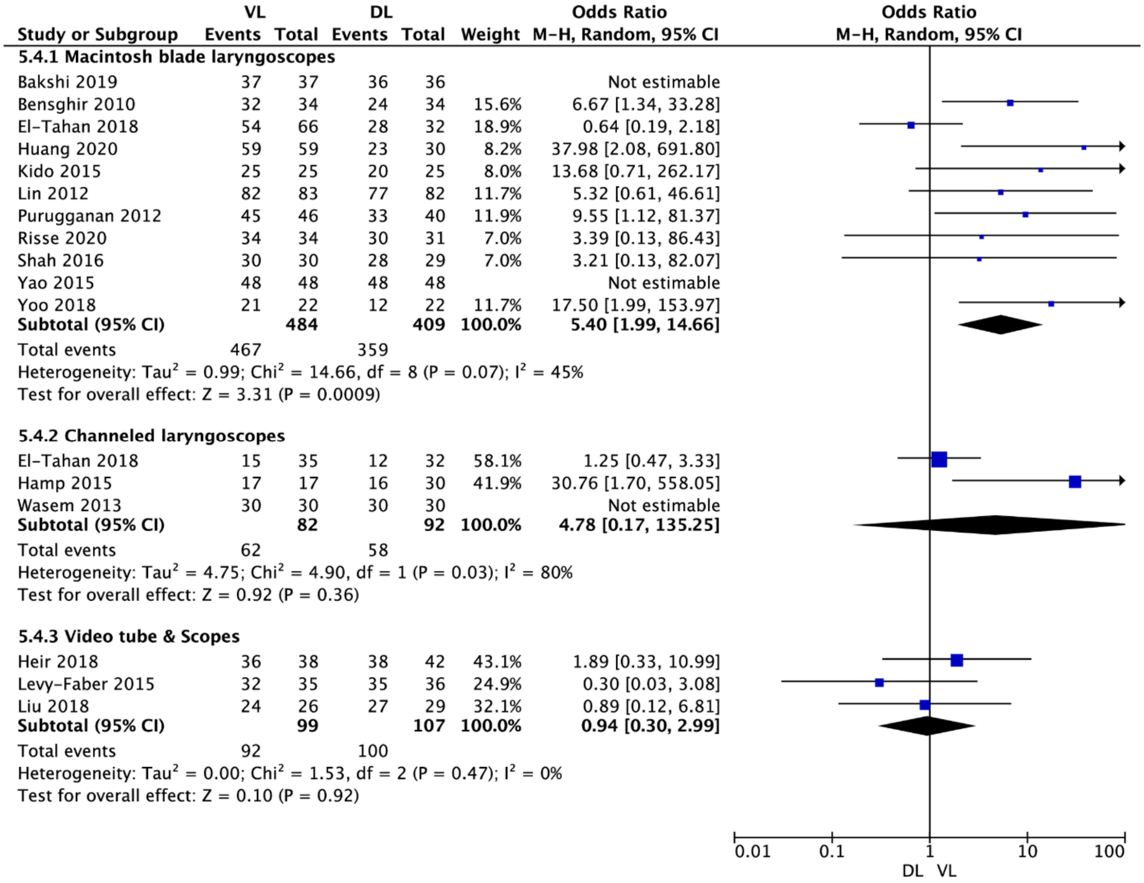

Figure S3. Forest plot of Cormack-Lehane 1 or 2 grade in video-laryngoscope and direct-laryngoscope groups. The center of each square represents the weighted odds ratios for individual trials, and the corresponding horizontal line stands for a 95% confidence interval. The diamonds represent pooled results. Legend: CI = confidence interval; DL = direct-laryngoscope; MD = mean difference; VL = video-laryngoscope.

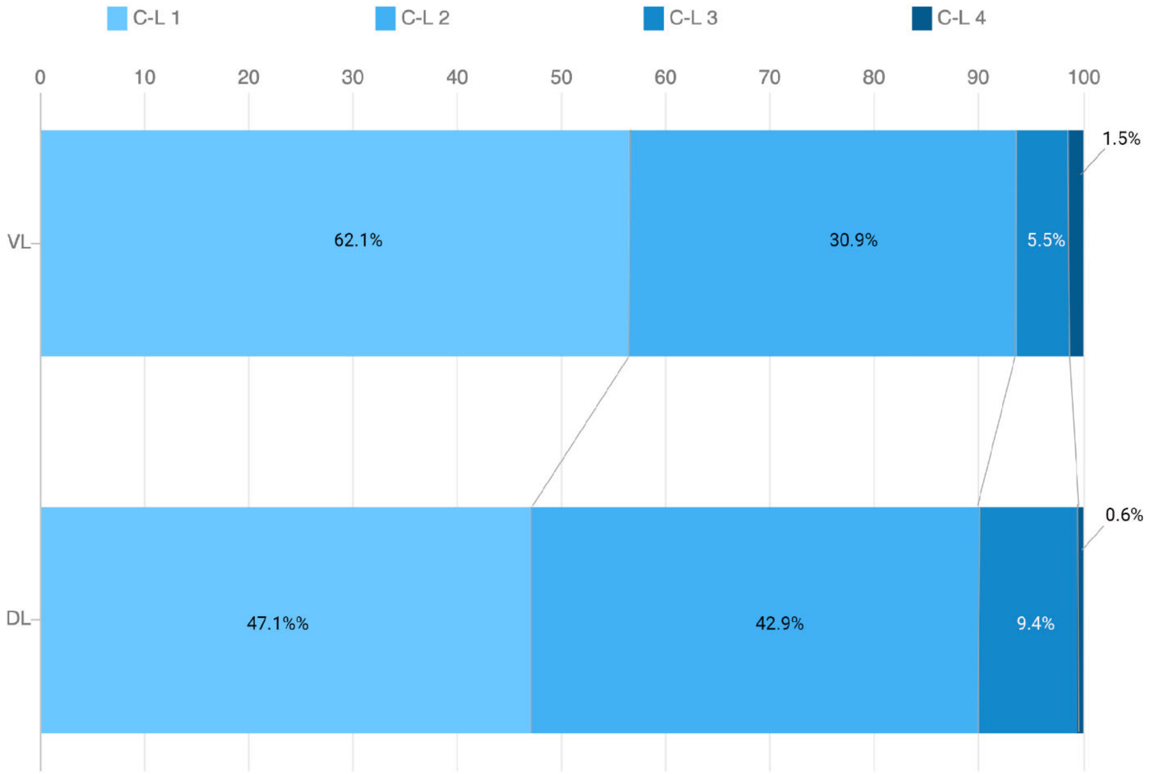

Figure S4. Distribution of glottis visualization in Cormack-Lehane grade among 1,500 patients .

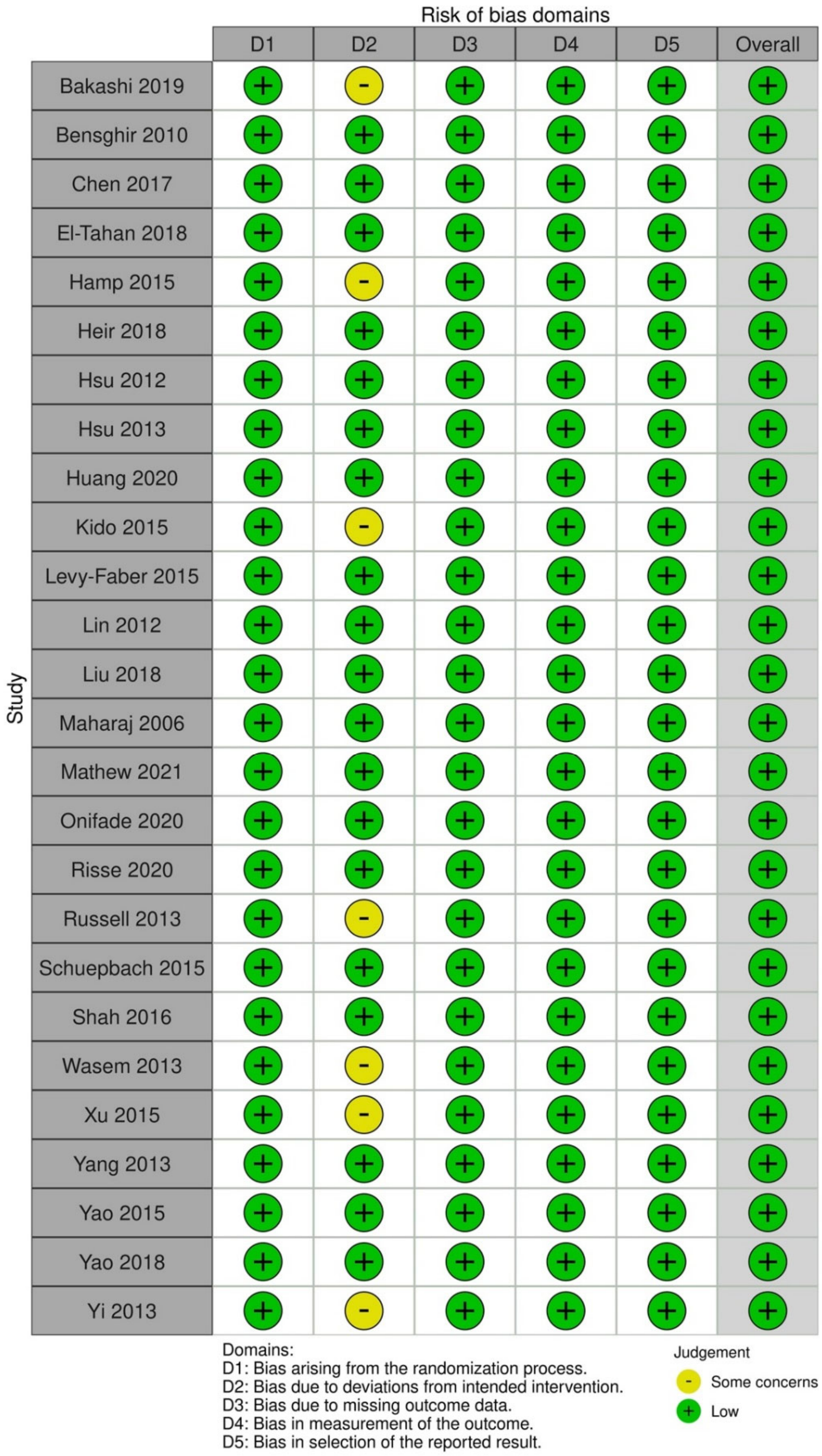

Figure S5. A summary table of review authors' judgements for each risk of bias item for each randomized study.

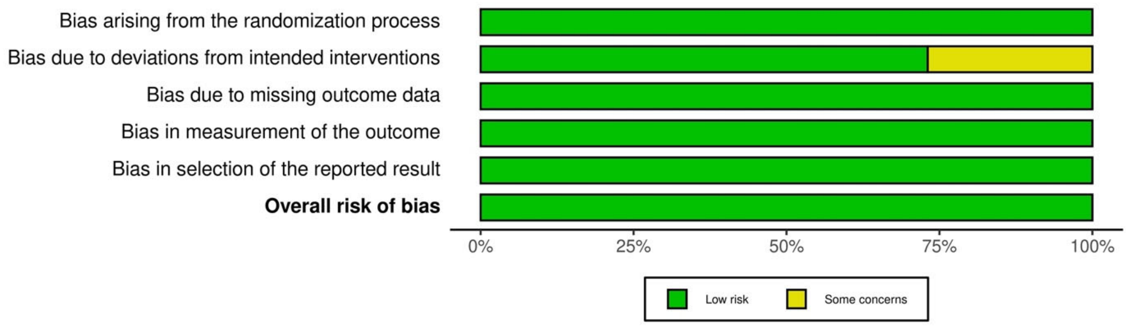

Figure S6. A plot of the distribution of review authors' judgements across randomized studies for each risk of bias item.
